# A Circular Fitting Clutter Suppression Algorithm Based on ISAC for Low Altitude UAVs

**DOI:** 10.3390/s25206285

**Published:** 2025-10-10

**Authors:** Qi Liu, Meng Song, Jinghan Yu, Peng Liang, Ti Wang, Chuanxin Zeng, Zhibin Zhang, Yibo Gao, Liu Liu

**Affiliations:** 1 China Unicom Smart City Research Institute, Beijing 100048, China; liuqi49@chinaunicom.cn (Q.L.); songmeng@chinaunicom.cn (M.S.); liangp@chinaunicom.cn (P.L.); wangti2@chinaunicom.cn (T.W.); zengxc12@chinaunicom.cn (C.Z.); zhangzb171@chinaunicom.cn (Z.Z.); 2 Software Institute, Nanjing University, Nanjing 210093, China; 241098114@smail.nju.edu.cn; 3 School of Electronic and Information Engineering, Beijing Jiaotong University, Beijing 100044, China; 24110060@bjtu.edu.cn

**Keywords:** ISAC, circular fitting algorithm, clutter suppression, low-altitude UAV

## Abstract

During the perception process of low-altitude unmanned aerial vehicles (UAVs), interference from strong static clutter generated by the ground and buildings is inevitable. To effectively reduce the interference from static clutter during the perception process, clutter suppression algorithms such as Moving Target Indicator (MTI) have been developed. However, existing algorithms have problems such as residual clutter interference and high computational complexity. To solve the above problem, this paper proposes a circular fitting clutter suppression algorithm based on the integrated communication and perception system. This method can suppress static clutter using the circular fitting algorithm by leveraging different OFDM symbols on subcarriers based on the OFDM echo channel characteristics of drone targets and static environmental interference. Simulation results show that this method can effectively suppress static clutter and significantly improve the distinguishability of the range-Doppler (RD) spectrum of dynamic targets. In addition, an energy ratio is proposed to quantitatively compare the clutter suppression effects of various algorithms. The method in this paper, especially in the perception performance of low-speed group targets, outperforms existing methods and can solve the problem of interference from the static clutter environment to the perception of dynamic targets in existing technologies.

## 1. Introduction

As a typical carrier of low-altitude economy, low-altitude UAVs are being widely used in urban distribution, inspection, agricultural monitoring, security, and other fields by virtue of their advantages of strong line-of-sight, wide coverage, and easy deployment. With the wide application of UAVs in the low-altitude field, how to realize high-precision and real-time supervision of UAVs in the low-altitude airspace has become an urgent problem to be solved. To meet this challenge, 3GPP is actively promoting the development of integrated sensing and communication (ISAC)-related standards. In the Rel-18 phase, the SA1 WG has carried out several use case studies, including UAV trajectory tracking, factory positioning, home security, highway intrusion detection, etc.; in the Rel-19 phase, the RAN1 WG has further promoted ISAC channel modeling, focusing on the modeling and evaluation of six categories of typical targets, including low-altitude UAVs, indoor/outdoor pedestrians, vehicles, and automated guided vehicles [1].

ISAC has emerged as an important development direction in the field of wireless communication, achieving the synergy of communication and sensing functions by sharing spectrum and hardware resources, which shows the advantages of high spectrum utilization and low hardware cost. This technology uses the reflected echo of communication signals for target detection and tracking, which is expected to be applied in the 5G-A and 6G [2]. Its dual capability to deliver both communication and high-precision sensing in scenarios like intelligent transportation systems, smart cities, vehicle-road coordination, and low-altitude drone surveillance positions it as a cornerstone of future ubiquitous IoT infrastructure and low-altitude economic ecosystems [3]. In order to optimize the sensing performance of the ISAC system, researchers have proposed various system design schemes, such as array and spherical wave [4,5,6].

Although the ISAC technology has broad prospects, its perception performance is severely restricted in actual deployment, especially in complex environments such as low-altitude urban areas. A core bottleneck problem is the strong static clutter interference. The strong echo signals from static environments such as the ground and buildings often drown out the weak dynamic echoes reflected by UAVs (especially low-speed and small UAVs), resulting in failed target detection or reduced trajectory tracking accuracy [7,8].

In response to the above issues. This paper proposes a circular fitting clutter suppression algorithm for low-altitude UAV scenarios, which can effectively suppress static clutter to achieve UAV sensing, especially low-speed target sensing. The method based on the existing MIMO-OFDM architecture is easy to deploy. The base station transmits the sensing detection signal, which is scattered by the UAV target and static clutter interference to form an echo signal received by the base station. In addition, the method shown in this paper can be extended to the scenario of trajectory fusion verification for remote identification (RID) of UAVs in Hangzhou. By designing an efficient echo signal processing process, the strong static clutter interference is suppressed so as to improve the sensing capability of dynamic targets such as UAV targets, which can provide technical support for the future city-level low-altitude UAV regulatory system.

## 2. Related Work

To cope with clutter interference, researchers mainly seek solutions from two aspects, signal processing and perception map modeling, which are described as follows:

(1) Signal processing level. Existing methods mainly enhance the dynamic target signal by identifying and removing the static clutter components. Echo signals are usually modeled as a superposition of static environmental clutter and dynamic target echoes, where the phase of static clutter remains constant within multiple OFDM symbols, while the phase of dynamic targets shows periodic variations with their radial velocity. Ref. [9] proposes a signal processing algorithm that aims to maximally eliminate clutter echoes from radar images by searching for the amplitude and distance parameters of each clutter scatterer in space and subtracting the clutter component from the original signal matrix before performing the Fast Fourier Transform computation. Ref. [10] proposes an OFDM echo processing method that combines the ambiguity function to achieve echo enhancement and anti-clutter optimization for low-altitude targets, which is especially suitable for weak motion target detection. Ref. [11] analyzes the statistical characteristics of radar clutter. Aiming at the blind speed problem of traditional MTI filters, a clutter suppression method based on staggered MTI is proposed. Ref. [12] proposes the design of multiple communication beams that can communicate with the user in both directions, as well as perception beams that can scan the space in all directions, in order to achieve perception under clutter interference in dynamic target environments. Ref. [13] proposes a perception algorithm based on clutter suppression that uses a 2D inverse Fast Fourier Transform algorithm for distance and speed estimation and a 2D multi-signal classification algorithm for angle of arrival estimation to effectively eliminate clutter interference. Ref. [14] proposes a practical integrated sensing and communication framework and constructs a hybrid sensing channel model that includes static environments and dynamic targets, which can sense dynamic targets in cluttered environments and guarantee the user communication quality at the same time. Considering the influence of non-side-looking airborne radar clutter dispersion on Space-Time Adaptive Processing (STAP), an efficient adaptive angular Doppler compensation method is proposed to improve the clutter suppression performance [15]. Ref. [16] uses planar adaptive angular Doppler compensation to significantly reduce on-board clutter interference.

(2) Perceptual map modeling. Researchers are committed to improving the quality and resolution of range-Doppler maps. Ref. [17] proposes an innovative waveform design method, Max-Aperture Radar Slicing (MaRS), with a novel processing method based on angular-Doppler plots, which significantly improves the ability of weak targets to separate from strong clutter and is a key support scheme for sensing scenes in complex environments such as low-altitude UAVs and urban monitoring. Ref. [18] proposes a low-complexity super-resolution range-Doppler map (RDM) modeling algorithm, which can effectively improve the applicability of OFDM in dynamic scenes. Ref. [19] systematically combs the application of deep learning in RDM image recognition, using deep learning to extract static and dynamic targets from echo maps to improve perceptual robustness. Ref. [20] proposes a model for resolving distance ambiguity by random logarithmic frequency offset, which significantly improves the estimation accuracy and resolution and facilitates more effective multi-target detection. Ref. [21] proposes an adaptive sea clutter suppression method based on meta-learning. This method takes the range-Doppler spectrum of sea clutter as a multi-channel input into the autoencoder, and uses unsupervised reconstruction as a proxy task to perform meta-training on the base model.

## 3. System Model

**Figure 1:**
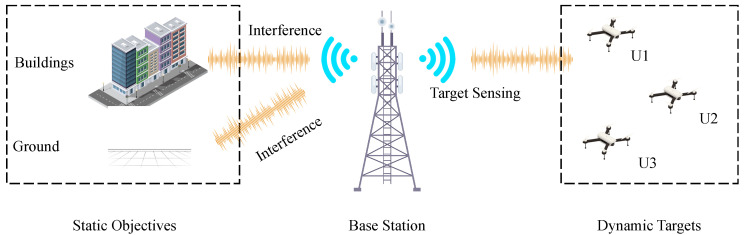
Base station sensing system model.

This section introduces the overall system model, which consists of the base station, ground, buildings, and low-altitude UAVs, with the base station transmitting OFDM signals, combined with beamforming to cover the sensing space. The model performs target sensing by using the reflected echoes of the communication signal. During the sensing process, the low-altitude UAVs generate echo signals, while the static environmental clutter caused by the ground and buildings generates strong echo signals that mask the weak signals of the UAV target. The base station sensing system model is shown in Figure 1.

The system is designed as a broadband massive MIMO system using OFDM modulation operating in the millimeter wave band. Based on an ISAC base station configured with transmitting and receiving arrays for beamforming and spatial beam scanning. The base station transmits a beam covering said sensing space in a beam scanning manner.

**Figure 2:**
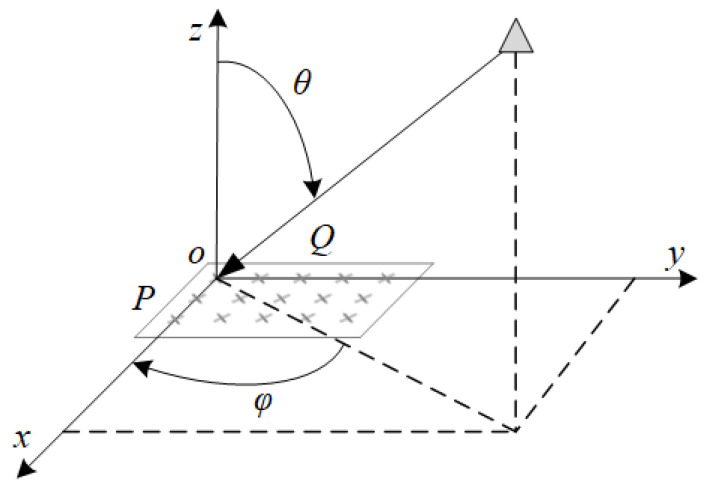
Uniform plane array model.

The base station is equipped with two Uniform Planar Arrays (UPAs) as the transmitting and receiving arrays, which are two-dimensional rectangular arrays consisting of a large number of antenna array elements with the numbers of antennas being $N_{t}$ and $N_{r}$, respectively, and the spacing between antennas being $d=\frac{\lambda }{2}=\frac{c}{2f}$. Here, λ represents the wavelength of the signal, and the reference antenna units are located at the origin of the coordinate system. By precisely controlling the phase and amplitude of each antenna element, the UPA is able to achieve complex beam shaping and spatial information processing. The UPA model is shown in Figure 2.

The base station transmits OFDM signals containing *M* symbols, employing *N* subcarriers with minimum and interval frequencies of $f_{0}$ and Δf, respectively. The frequency of the *n*th subcarrier is $f_{n}=f_{0}+\left(n-1\right)\Delta f$, where n=1,…,N. The UAVs are selected as dynamic targets in the model, and the base station monitors the UAVs using *M* consecutive OFDM symbols at a single time, with a time interval of each OFDM symbol set to $T_{s}=\frac{1}{\Delta f}$.

The spatial Cartesian coordinate system is used to define the location of the UAV targets and the static environmental interference, and the spherical coordinate system is used for the channel analysis, where the radial distance r>0, the azimuth angle $\theta \in [0^{\circ },180^{\circ }]$, and the elevation angle $\phi \in [-90^{\circ },90^{\circ }]$. Assuming that there are *P* UAV targets within the service range of the base station, the position and radial speed of the *p*th UAV target are $\left(r_{p},\theta_{p},\phi_{p}\right)$ and $v_{p}$, respectively, where p=1,…,P. There are *Q* static environmental clutter interferences within the service range of the base station, and the location of the *q*th static environmental interference is $\left(r_{q},\theta_{q},\phi_{q}\right)$, where q=1,…,Q.

## 4. Basic Model of Echo Channel

The base station transmits a signal that is scattered by the targets and clutter units to form an echo channel matrix, which is superimposed on the noise to generate a signal tensor for subsequent sensing of the dynamic targets. The echo channel model for this sensing system contains *P* UAV targets and *Q* static clutter. The frequency domain echo channel matrix of the *p*th UAV target at the *m*th symbol of the *n*th subcarrier is

(1) $$H_{m,n}^{p}=\alpha_{p}e^{j2\pi f_{0}\frac{2\nu_{p}}{c}mT_{s}}e^{-j2\pi f_{n}\frac{2r_{p}}{c}}a_{r}\left(\Psi_{p},\Omega_{p}\right)a_{t}^{T}\left(\Psi_{p},\Omega_{p}\right),$$

where m=1,…,M, n=1,…,N, $\alpha_{p}$ is the echo channel fading factor of the *p*th UAV target, *c* is the speed of light, and $a_{r}\left(\Psi_{p},\Omega_{p}\right)$ and $a_{t}\left(\Psi_{p},\Omega_{p}\right)$ are the array orientation vectors of the receiving and transmitting arrays pointing towards the spatial domain direction of the *p*th UAV target, respectively. The dimension of the UPA is set to be $N_{t}\times N_{r}$. The angle of a certain signal is set to be (Ψ,Ω), where Ψ and Ω denote its azimuth and pitch angle, respectively. The conversion relationship between the spatial domain direction (Ψ,Ω) and the physical direction (θ,ϕ) is Ψ=cosϕcosθ and Ω=sinϕ. The phase difference between the (a,b)th UPA antenna array element and the reference array element is

(2) $$\varphi_{a,b}\left(\Psi ,\Omega \right)=e^{-j2\pi d(a\cos\Psi \sin\Omega +b\sin\Psi \sin\Omega )/\lambda },$$

where $a=0,\ldots ,N_{t}-1$, $b=0,\ldots ,N_{r}-1$. The array orientation vector of the signal is

(3) $$a\left(\Psi ,\Omega \right)={\left[\varphi_{a,b}\left(\Psi ,\Omega \right)\right]}_{a=0,\ldots ,N_{t}-1;b=0,\ldots ,N_{r}-1}^{T}.$$

Similarly, the frequency domain echo channel matrix at the *m*th symbol on the *n*th subcarrier of the *q*th static environmental interference is

(4) $$H_{m,n}^{\prime q}=\beta_{q}e^{-j2\pi f_{n}\frac{2r_{q}}{c}}a_{r}\left(\Psi_{q},\Omega_{q}\right)a_{t}^{T}\left(\Psi_{q},\Omega_{q}\right),$$

where $\beta_{q}$ is the echo channel fading factor of the *q*th static ambient interference. $a_{r}\left(\Psi_{q},\Omega_{q}\right)$ and $a_{t}\left(\Psi_{q},\Omega_{q}\right)$ are the array guidance vectors of the receiving and transmitting arrays pointing in the direction of the *q*th static clutter spatial domain, respectively. Therefore, the frequency domain echo channel matrix of all static clutter and UAV targets at the *m*th symbol of the *n*th subcarrier is

(5) $$\begin{array}{cc} H_{m,n} & =\sum_{p=1}^{P}H_{m,n}^{p}+\sum_{q=1}^{Q}H_{m,n}^{\prime q}\\ & =\sum_{p=1}^{P}\alpha_{p}e^{j2\pi f_{0}\frac{2\nu_{p}}{c}mT_{z}}e^{-j2\pi f_{n}\frac{2r_{p}}{c}}a_{r}\left(\Psi_{p},\Omega_{p}\right)a_{r}^{T}\left(\Psi_{p},\Omega_{p}\right)\\ & +\sum_{q=1}^{Q}\beta_{q}e^{-j2\pi f_{n}\frac{2r_{q}}{c}}a_{r}\left(\Psi_{q},\Omega_{q}\right)a_{t}^{T}\left(\Psi_{q},\Omega_{q}\right) \end{array}.$$

As shown in Equation (5), take the scenario where there is only one UAV and one clutter source in the environment as an example. The base station uses the transmitting array and the receiving array for beam assignment and spatial beam scanning to obtain the echo signals in the environment. Then the frequency-domain echo channel matrix is only composed of $H_{m,n}^{1}$ and $H_{m,n}^{\prime 1}$. Assuming that the spatial domain direction of the beam scanning of the base station in the two-dimensional angular space is $\left(\Psi_{s},\Omega_{s}\right)$, at this time, the transmitting beam assignment vector and the receiving beam assignment vector of the base station are

(6) $$w_{t}=\sqrt{\frac{P_{s}}{N_{t}}}a_{t}\left(\Psi_{s},\Omega_{s}\right).$$

(7) $$w_{r}=\sqrt{\frac{1}{N_{r}}}a_{r}\left(\Psi_{s},\Omega_{s}\right).$$

Then, the echo signal on the *m*th symbol of the *n*th subcarrier received by the base station during beam scanning is

(8) $$y_{m,n}=w_{r}^{H}H_{m,n}w_{t}^{*}+noise_{m,n},$$

where $noise_{m,n}$ represents zero-mean additive complex Gaussian noise. After completing beam scanning, the base station concatenates the echo signals $y_{m,n}$ generated from all subcarriers and all OFDM symbols to form an echo signal matrix Y. Subsequent signal processing of this matrix enables target perception.

## 5. Circle Fitting Suppression Algorithm

The circular fitting suppression algorithm reconstructs the static clutter phase position by analyzing the circular arc trajectory of the echo signal plane so as to eliminate the static environmental interference, extract the effective signal of the UAV target, and obtain the echo matrix after clutter suppression. This method involves taking the echo signal vectors of the UAV target and clutter obtained from each symbol of each subcarrier. The real part of the vector is used as the abscissa, and the imaginary part is used as the ordinate, both of which are placed in a two-dimensional complex plane. Then, circle fitting is performed on the points in the coordinate system to obtain the center of the fitted circle. Finally, the points in the two-dimensional complex plane are moved to the origin of the coordinate system along with the fitted center, thereby achieving clutter suppression.

**Figure 3:**
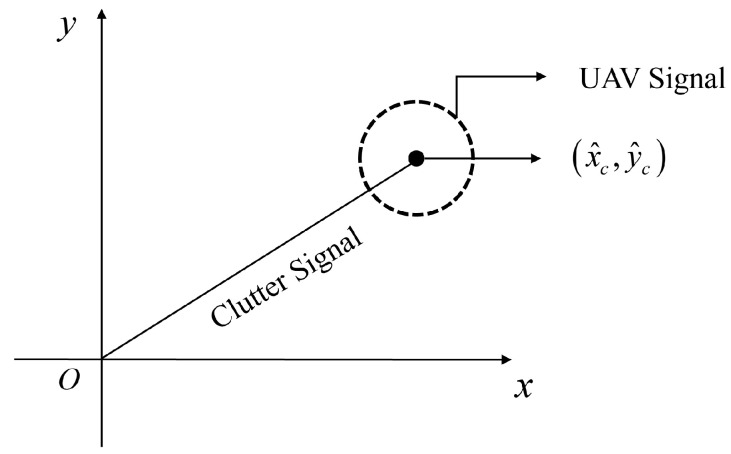
The schematic diagram of the circular fitting suppression algorithm.

Using the echo signal matrix Y to sense the UAV targets in the environment, the echo signal vector $y_{n}$ on the *n*th subcarrier in Y is extracted sequentially, which consists of complex numbers. Its expression is as follows:

(9) $$y_{n}=\left[\begin{array}{c} w_{r}^{H}H_{1,n}w_{t}^{*}+noise_{1,n}\\ w_{r}^{H}H_{2,n}w_{t}^{*}+noise_{2,n}\\ \vdots \\ w_{r}^{H}H_{m,n}w_{t}^{*}+noise_{m,n}\\ \vdots \\ w_{r}^{H}H_{M,n}w_{t}^{*}+noise_{M,n} \end{array}\right]=\left[\begin{array}{c} z_{1}\\ z_{2}\\ \vdots \\ z_{m}\\ \vdots \\ z_{M} \end{array}\right]=\left[\begin{array}{c} x_{1}+jy_{1}\\ x_{2}+jy_{2}\\ \vdots \\ x_{m}+jy_{m}\\ \vdots \\ x_{M}+jy_{M} \end{array}\right],$$

where the expression of $z_{m}$ is

(10) $$\begin{array}{cc} z_{m} & =w_{r}^{H}(\alpha_{p}e^{j2\pi f_{0}\frac{2\nu_{p}}{c}mT_{c}}e^{-j2\pi f_{n}\frac{2r_{p}}{c}}a_{r}\left(\Psi_{p},\Omega_{p}\right)a_{t}^{T}\left(\Psi_{p},\Omega_{p}\right)\\ & +\sum_{q=1}^{Q}\beta_{q}e^{-j2\pi f_{n}\frac{2r_{q}}{c}}a_{r}\left(\Psi_{q},\Omega_{q}\right)a_{t}^{T}\left(\Psi_{q},\Omega_{q}\right))w_{t}^{*} \end{array},$$

where m=1,…,M. Since $y_{n}$ contains multiple static environmental interference targets with constant phase within *M* OFDM symbols on the *n*th subcarrier and *p*th dynamic UAV targets with nearly constant amplitude but varying phase within *M* OFDM symbols. The phase variation in the UAV targets in the two-dimensional complex plane produces a circular arc centered on the static clutter interference vectors around the points where the static environmental interference phase does not vary the trajectory. The Schematic diagram of the circular fitting suppression algorithm is shown in Figure 3.

Define the generic equation of the complex plane circle as

(11) $${\left(x-x_{c}\right)}^{2}+{\left(y-y_{c}\right)}^{2}=R^{2},$$

where $\left(x_{c},y_{c}\right)$ represents the center of the circle and *R* represents the radius. The equation is expanded and linearized as

(12) $$x_{m}^{2}+y_{m}^{2}+Dx_{m}+Ey_{m}+F=0.$$

Among them, $D=-2x_{c}$, $E=-2y_{c}$, and $F=x_{c}^{2}+y_{c}^{2}-R^{2}$ are used to construct the overdetermined system and solve the parameters by the least squares method.

(13) $$\left(\hat{D},\hat{E},\hat{F}\right)=argmin∥\begin{array}{ccc} x_{1} & y_{1} & 1\\ x_{2} & y_{2} & 1\\ \vdots & \vdots & \vdots \\ x_{M} & y_{M} & 1 \end{array}∥\left[\begin{array}{c} D\\ E\\ F \end{array}\right]+\left[\begin{array}{c} x_{1}^{2}+y_{1}^{2}\\ x_{2}^{2}+y_{2}^{2}\\ \vdots \\ x_{M}^{2}+y_{M}^{2} \end{array}\right].$$

Through Equation (13), we can obtain the parameters $\hat{D}$, $\hat{E}$, and $\hat{F}$, and calculate the center of the fitting circle. The analytical solution for the center coordinates and radius is obtained as

(14) $$\left\{\begin{array}{c} {\hat{x}}_{c}=-\hat{D}/2\\ {\hat{y}}_{c}=-\hat{E}/2\\ \hat{R}=\sqrt{{\hat{x}}_{c}^{2}+{\hat{y}}_{c}^{2}-\hat{F}} \end{array}\right..$$

Take *M* OFDM symbols of $y_{m}$ as input for circle fitting to obtain the static clutter interference vector whose value is the center of the fitted circle $z^{s}=\left({\hat{x}}_{c},{\hat{y}}_{c}\right)$, and reconstruct the center of the static interference signal $y^{s}$ as

(15) $$y^{s}=\left[\begin{array}{c} z^{s}\\ z^{s}\\ \vdots \\ z^{s} \end{array}\right]\in C^{M\times 1}$$

Based on this, the effective echo signal of the UAV dynamic targets $y^{d}$ can be obtained as

(16) $$y^{d}=y-y^{s}=\left[\begin{array}{c} z_{1}-z^{s}\\ z_{2}-z^{s}\\ \vdots \\ z_{M}-z^{s} \end{array}\right]\in C^{M\times 1},$$

where y is the vector of all symbols in the current subcarrier. Then all the subcarriers are used for clutter removal by circular fitting. $y_{n}^{d}$ represents all the symbol vectors of the *n*th subcarrier, and $y_{n}^{d}$ are reassembled into the echo signal matrix $Y^{d}$, that is

(17) $$Y^{d}=\left[y_{1}^{d}y_{2}^{d}\ldots y_{N}^{d}\right]\in C^{M\times N}.$$

The static clutter interference is a fixed point in the complex plane around which the dynamic targets vary in phase. Therefore, the obtained center of the circle represents the phase quantity of the static clutter interference, and placing it at zero can achieve static clutter suppression.

## 6. Simulation and Verification

This section presents simulation verification based on the model above. First, parameters such as the distance of the UAV targets and static clutter are configured. The echo signal matrix is then obtained according to the echo channel model. Subsequently, a circle-fitting suppression algorithm is employed to eliminate static clutter. The Table 1 outlines the relevant simulation parameter settings.

### 6.1. Verification of Clutter Suppression Effect

**Figure 4:**
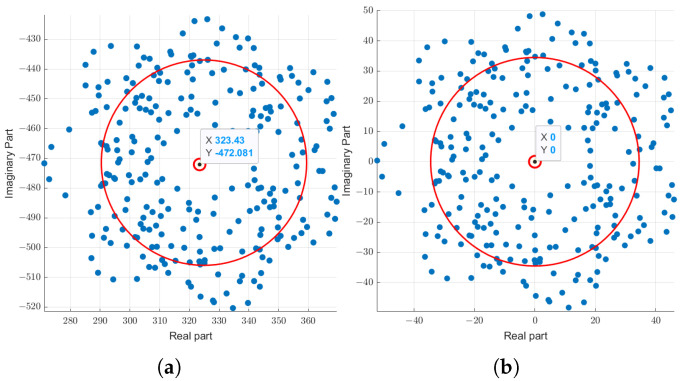
Echo signal vectors before and after clutter suppression. (**a**) Original echo signal with fitting circle. (**b**) Echo signal after clutter suppression.

To verify the effectiveness of the clutter suppression of this paper’s algorithm, this paper constructs a simulation scene containing 3 UAV targets and 10 static clutter objects. The UAV target echo channel fading factors set by Table 1 are 0.7, 1, and 0.8, respectively, which are significantly lower than the static clutter echo channel fading factor of 10, simulating a strong clutter environment. The method of this paper is used to process the original echo signal to eliminate the static clutter interference. Figure 4 shows the adjustment of a subcarrier in the clutter suppression process.

The point in Figure 4a is the original echo signal vector of a subcarrier before carrier clutter suppression, and the circle fitted according to this vector is the large circle in the figure, and the small circle in the center of the figure is the center of the fitted circle. The return signal vector after clutter suppression can be obtained by placing the center of the circle at the origin, as shown in Figure 4b.

**Figure 5:**
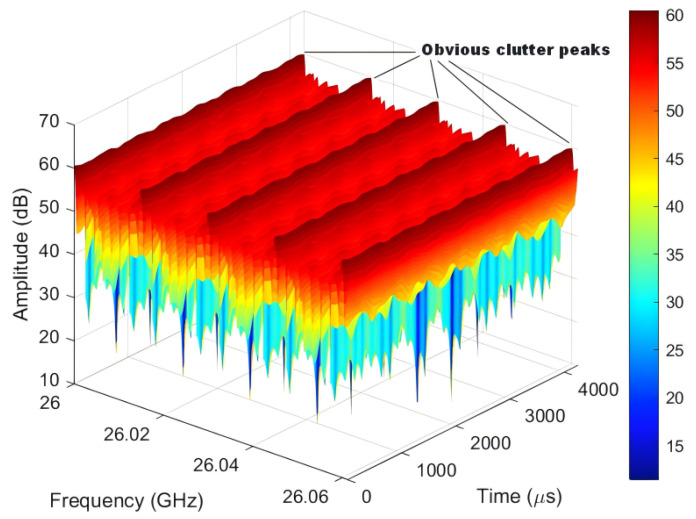
The original echo signal.

The original echo signal covering all subcarriers is then considered. The original echo signal consists of the UAV targets mixed with static clutter, as shown in Figure 5. It can be seen that in the case of the mixed effect of UAV targets and static clutter, the original echo signal covers the UAV target signals with lower amplitude and the static clutter signals with higher amplitude. The peaks of clutter signals can be clearly observed.

**Figure 6:**
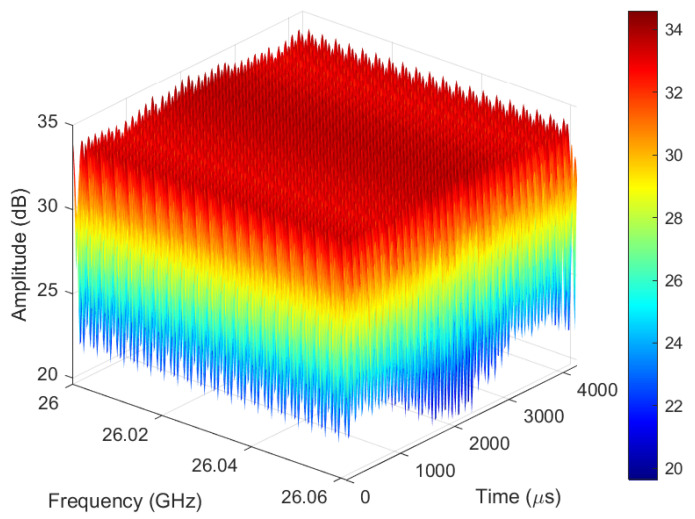
The suppressed echo signal.

Observe the suppressed echo signal as shown in Figure 6. After applying static clutter suppression using the circular fitting algorithm to the original echo signals, the effective echo signals of the UAV targets show a more periodic distribution than that before suppression, which reflects the modulation characteristics of the UAV motion process. Compared with Figure 5, the static clutter signal can no longer be clearly observed in Figure 6.

**Figure 7:**
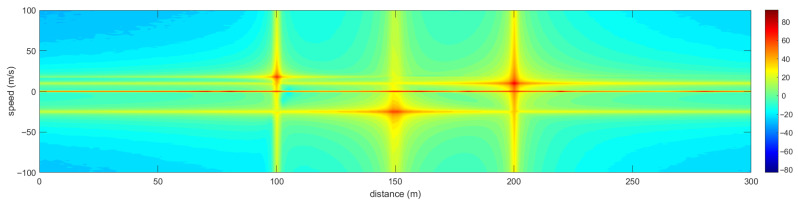
RD spectrum of the original echo signal.

In order to visualize the perception of the UAV targets, the original echo signal is directly processed by 2D Fast Fourier Transform (2D-FFT), and the range-Doppler (RD) spectrum of the signal can be obtained, as shown in Figure 7. It can be observed that the red part of the figure indicates the presence of UAV targets and strong static clutter. The red line with zero speed in the figure indicates the presence of significant static clutter peaks, and the non-zero speed indicates the UAV target peaks. At this time, the relevant information of the UAV targets cannot be extracted directly.

**Figure 8:**
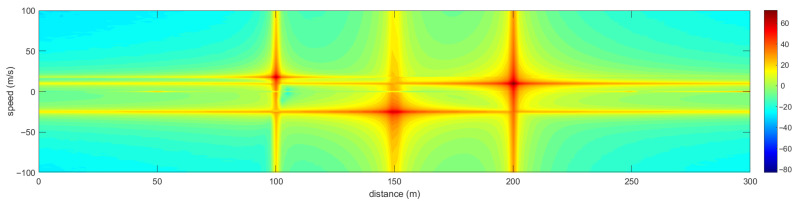
RD spectrum of the echo signal after clutter suppression.

The RD spectrum obtained by 2D Fast Fourier Transform (FFT) of the signal after clutter suppression is shown in Figure 8. The UAV targets to be acquired in the sensing space can be clearly observed, and the remaining three peaks correspond to the radial speeds and position information of the three UAV targets, respectively. The static clutter peaks with zero speed in the original echo signal of Figure 7 can no longer be observed, indicating that the static environmental clutter has been significantly suppressed at this time. We also tested the situation where the clutter and UAV power are similar, and the results are consistent with those shown in Figure 7, indicating that the method is robust.

### 6.2. Comparison of Existing Algorithms

The most typical existing static clutter suppression algorithms are Moving Target Indicator (MTI) and Zero-Speed Channel Zeroing. MTI uses clutter suppression filters to suppress clutter and improve the signal-to-noise ratio of the radar signal, which is conducive to the technology of moving target detection. The zero-speed channel zeroing method directly sets the zero-speed channel or the channel near zero-speed in the speed channel of the RD plot to zero after the 2D-FFT, and this operation means that stationary targets or low-speed targets will directly disappear from the R-V spectral matrix. In order to quantify the superiority of the method in this paper, the MTI and zero-speed channel zeroing method are applied to the low-altitude UAV sensing scenario. In addition, to quantitatively assess the target enhancement capability, the UAV targets are divided into three groups of UAVs with different speeds, namely, the low-speed group (≤1 m/s), the medium-speed group (1–5 m/s), and the high-speed group (>5 m/s), based on the existing low-altitude UAV motion speeds. The specific speed settings of each group of UAVs during the validation process are shown in Table 1. The energy ratio is then calculated to compare the advantages of each method. Here, the energy ratio is the ratio of the energy of the target at the distance-Doppler unit after clutter suppression to the energy of the target at the distance-Doppler unit in the original RD spectrum.

As shown in Table 2, the circular fitting clutter suppression method proposed in this paper outperforms the MTI in all speed conditions, and the energy ratios in low, medium and high speeds are improved by 41.85 dB, 25.82 dB and 12.90 dB, respectively, and the higher the speed is, the closer the suppression effects of the two methods are. In the middle- and high-speed conditions, the zero-speed channel zeroing method and our method are quite close to each other, and our method only improves 0.09 dB relative to the zero-speed channel zeroing method in the middle-speed condition. While in the high-speed condition, the two methods have the same suppression effect. In the low-speed condition, the method in this paper only improves by 0.58 dB compared with the meta-learning algorithm, while in the medium- and high-speed conditions, the method in this paper is slightly inferior to the meta-learning algorithm by about 3 dB. However, considering the algorithm complexity, the method in this paper is more complex than the meta learning algorithm.

However, at low speeds, the proposed method outperforms the other methods, and this gain proves that the proposed method has superior low-altitude UAV sensing capability in complex clutter environments and still has better sensing capability than other methods at low speeds.

### 6.3. Experimental Verification Scenario

The proposed method is based on MIMO-OFDM and beamforming technology, which can be deployed through existing base stations. The circular fitting clutter suppression technique has been verified in simulation to significantly improve the sensing capability of low-altitude UAV targets in complex environments. It will be able to carry out field tests with communication equipment manufacturers in the future and has been planned to be integrated into the next-generation communication sensing integration standard system. The method has a solid foundation and market prospect for rapid industrialization, with priority given to commercial applications in scenarios such as smart city security and low-altitude monitoring of key areas, and can be applied to the following test scenarios.

In the verification of RID trajectory fusion for UAVs in Hangzhou, the initial UAV RID data and the sensing data of the UAV from the 5G-A base station are fused, and the method of this paper can be applied to achieve a more accurate sensing of the UAV movement data in this sensing process.

**Figure 9:**
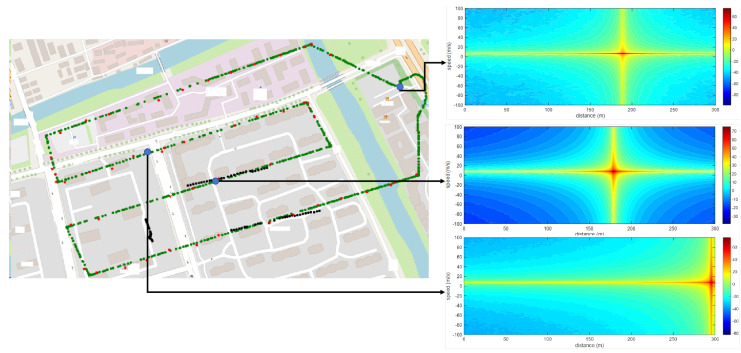
Trajectory map of UAV in Hangzhou.

As shown in Figure 9, the green dots in the figure are the initial UAV RID points, and the black dots are the UAV trajectory points sensed by the base station; by increasing the sensing data of the base station to the UAV, it is possible to make up for the missing area of the initial RID and refine the trajectory. Three blue dots are randomly extracted from the map, and three pictures on the right are obtained by using the method in this paper for UAV clutter suppression. It is shown that the method can effectively achieve clutter suppression.

## 7. Conclusions

In this paper, a clutter suppression algorithm based on circle fitting is proposed for the problem of degradation of low altitude UAV target sensing performance caused by static clutter interference in the ISAC system. The method effectively rejects static clutter interference by analyzing the return signal matrix of static clutter and UAV targets on OFDM subcarriers and reconstructing the return signal center using a circle fitting algorithm. Simulation results show that this method can significantly improve the RD spectral discrimination of dynamic targets in a strong static clutter environment, and effectively improve the sensing capability of dynamic targets such as UAVs.

By comparing energy ratios with existing methods, our approach demonstrates superior performance in clutter suppression while preserving target energy. Specifically, compared to MTI methods, it achieves a 41.85 dB higher energy ratio when detecting a low-speed group, thereby enhancing detection capability. In addition, this algorithm is based on the MIMO-OFDM communication architecture, which is easy to integrate into the existing base station system and provides a practical solution for low-altitude UAV monitoring and other scenarios. While the UAV parameters used in this study are predefined and assumed, other algorithm constraints, including environmental conditions, flight speed ranges, and computational requirements, will be investigated to further optimize the effectiveness of suppression.

## Figures and Tables

**Table 1:** Parameter settings.

Parameter	Setting
Minimum subcarrier frequency	26 GHz
Speed of light	$3\times 10^{8}$ m/s
Number of OFDM symbols	256
Number of subcarriers	1024
Subcarrier frequency interval	60 KHz
Transmitter antenna array parameters	64
Receiver antenna array parameters	64
Number of UAV targets	3
Radial distances of UAV targets	100 m, 150 m, 200 m
Radial speeds of UAV targets	8 m/s, −10 m/s, 5 m/s
Azimuths of UAV targets	$15^{\circ },10^{\circ },20^{\circ }$
Elevation angles of UAV targets	$5^{\circ },-3^{\circ },8^{\circ }$
Echo channel fading factors of UAV targets	0.7, 1, 0.8
Number of static clutter	10
Radial distances of static clutter	70 m, 230 m, 190 m, 110 m, 280 m, 80 m, 180 m, 220 m, 120 m, 160 m
azimuths of static clutter	$15^{\circ },25^{\circ },35^{\circ },40^{\circ },10^{\circ },20^{\circ },30^{\circ },5^{\circ },40^{\circ },30^{\circ }$
Elevation angles of static clutter	$-8^{\circ },12^{\circ },-15^{\circ },5^{\circ },10^{\circ },-6^{\circ },7^{\circ },9^{\circ },-13^{\circ },2^{\circ }$
Echo channel fading factors of static clutter	10
Signal-to-noise ratio	4 dB
Radial speeds of low-speed group UAVs	0.6 m/s, −1 m/s, 0.5 m/s
Radial speeds of medium-speed group UAVs	3 m/s, 1.5 m/s, −2 m/s
Radial speeds of high-speed group UAVs	10 m/s, −25 m/s, 18 m/s

**Table 2:** Energy comparison of different algorithms.

Signal Type	Low-Speed Group Energy Ratio (dB)	Medium-Speed Group Energy Ratio (dB)	High-Speed Group Energy Ratio (dB)
Original RD spectrum	0	0	0
MTI	−61.10	−47.94	−35.06
Zero speed channel zeroing	−25.04	−22.21	−22.16
Meta Learning	−19.83	−18.46	−18.47
Method in this paper	−19.25	−22.12	−22.16

## Data Availability

The data presented in this study is available upon request from the corresponding author.

## Abbreviations

The following abbreviations are used in this manuscript:

UAV	Unmanned Aerial Vehicle
MTI	Moving Target Indicator
RD	Range-Doppler
ISAC	Integrated Sensing and Communication
5G-A	5G-Advanced
6G	6-Generation
IoT	Internet of Things
MIMO	Multi-Input Multi-Output
OFDM	Orthogonal Frequency Division Multiplexing
RID	Remote Identification
UPA	Uniform Planar Array
